# Mesenchymal Traits as an Intrinsic Feature of Undifferentiated Cells

**DOI:** 10.3390/jdb13010001

**Published:** 2024-12-24

**Authors:** Mirco Galiè

**Affiliations:** Department of Neuroscience, Biomedicine and Movement—Sec. Anatomy and Histology, University of Verona, Via Le Grazie 8, 37134 Verona, Italy; mirco.galie@univr.it; Tel.: +39-045-8027681

**Keywords:** mesenchymal, epithelial, stem, embryo, cancer

## Abstract

Since its first conceptualization over a century ago, the mesenchymal phenotype has traditionally been viewed as either a transient phase between successive epithelial stages or as a feature of cell types primarily devoted to structural support. However, recent findings in cancer research challenge this limited view, demonstrating that mesenchymal traits and hybrid mesenchymal/epithelial states can mark cancer cells with stem cell properties. By analyzing publicly available single-cell transcriptome datasets from early embryonic stages and adult tissues, this study aims to extend this concept beyond pathological contexts, suggesting that a partial or fully mesenchymal phenotype may represent the morphological expression of undifferentiated and multipotent states in both the developing embryo and adult organs.

## 1. Introduction

Epithelium and mesenchyme are regarded as the basic tissue types in vertebrates, as they predominate in the early stages of embryogenesis and give rise to all other tissues (connective, neural, and muscle) only later during morphogenesis. Traditionally, the distinction between epithelial and mesenchymal phenotypes is based on morphological criteria and a few molecular determinants, which have been enhanced by advances in molecular investigations [1,2,3]. Essentially, epithelial cells are round to polygonal in shape, exhibit apico-basal polarity, are connected through adherens and tight junctions, and can be identified by the expression of proteins that mediate cell-to-cell contacts, such as E-Cadherin, alpha-/beta-Catenin, and Cytokeratin (mainly types 8 and 18). Mesenchymal cells have an irregular shape, lack clear cell polarization, are loosely connected to neighboring cells, exhibit an invasive and migratory phenotype, and display positivity for Vimentin, N-Cadherin, P-Cadherin, and Fibronectin. This phenotype is driven by molecular programs mainly regulated by transcription factors such as zinc-finger E-box-binding homeobox factors ZEB1 and ZEB2, SNAIL, SLUG, and basic helix–loop–helix factors TWIST1 and TWIST2.

The transition from epithelial to mesenchymal state (EMT) and its reverse (MET) are fundamental events in embryonic development, while the reactivation of the EMT program in adults has long been recognized as a hallmark of cancer progression. However, recent advances in cancer biology have identified intermediate states displaying a mix of epithelial and mesenchymal features (hybrid E/M or quasi-mesenchymal), which challenge the traditional dichotomy between epithelial and mesenchymal phenotypes. These hybrid states characterize cancer cells with “stem cell properties” and can be isolated from advanced carcinomas or generated through experimentally induced EMT [4,5,6,7]. Notably, these hybrid states have been identified as pathological epithelial derivatives resulting from partial or incomplete EMT. However, there is evidence that they are constitutive features of “normal” stem cell populations, at least in the breast [4]. Additionally, a recent study reported the occurrence of a hybrid E/M state during organogenesis [8].

This raises the question of whether and how the hybrid E/M phenotype might represent a general paradigm of intrinsic plasticity and stem cell identity, potentially shifting toward fully epithelial or mesenchymal states depending on context and differentiation. To begin addressing this question, this paper explores the transcriptional expression of a collection of epithelial and mesenchymal genes in multiple, partially overlapping publicly available single-cell RNA-seq datasets spanning developmental stages from the totipotent zygote to adult precursor cells. The results provide a summary map of the evolution of epithelial and mesenchymal states through development, showing that the embryonic stages preceding epiblast formation (pre-implantation embryo) display traits of a mesenchymal phenotype and that hybrid E/M states persist throughout development as hallmarks of precursor cells from all three germ layers in adults.

## 2. Materials and Methods

### 2.1. Dataset Resources

Single-cell RNA-seq normalized datasets from Xue et al. [9], Yan et al. [10], and Mittnensweig et al. [11] were obtained from the Gene Expression Omnibus (GEO) under accession numbers GSE44183, GSE36552, and GSE169210, respectively. The RNA-seq normalized dataset from Petropoulos [12] was downloaded from the ArrayExpress database under accession E-MTAB-3929. Eraslan’s single-nucleus RNA-seq normalized dataset [13] was obtained from the GTEx Portal (https://gtexportal.org/home/) as file “GTEx_8_tissues_snRNAseq_atlas_071421.public_obs.h5ad”.

Processed data were downloaded as provided by respective authors, without any additional normalization step.

### 2.2. Heatmap and Hierarchical Clustering

Heatmaps were generated using Morpheus software, freely available at https://software.broadinstitute.org/morpheus/. Hierarchical clustering of genes and samples was performed based on Pearson correlation and average linkage.

### 2.3. PCA

Principal Component Analysis (PCA) of the GTEx-derived dataset was performed using the R packages Plotly, RColorBrewer, and FactoMineR. PCA yielded five dimensions. A k-means algorithm (with two clusters) was used to classify samples based on PCA results. A cell-type-specific color gradient from violet to black was assigned to each sample according to the mean k-means factor value for all samples of the same cell type.

### 2.4. Scatterplots and Correlation Analysis

Three-dimensional scatterplots and correlation statistics of the GTEx-derived dataset were generated using the R packages Plotly, Dplyr, RColorBrewer, and Htmlwidgets. Linear regression fitting was based on Ordinary Least Squares (OLS) to estimate the relationship between mean MES and mean EPITH as independent variables, with mean STEM (organ-specific) as the dependent variable.

#### Correlation Matrix Analysis

To generate correlation matrices, processed data were loaded as an .h5ad file using the Zellkonverter R package (https://bioconductor.org/packages/release/bioc/html/zellkonverter.html v.3.2) and analyzed using the SingleCellExperiment, Matrix, dplyr, tibble, ComplexHeatmap, and circlize packages according to the following workflow:

1. The mean expression values of mesenchymal (mean MES) and epithelial (mean EPITH) genes, as well as their ratio (MES/EPITH ratio), were calculated for each sample.
2. The mean MES, mean EPITH, and MES/EPITH ratio were added to the metadata for each sample.
3. The expression data (either the whole transcriptome or a restricted MES and EPITH gene dataset) were reduced for subsequent analysis by collapsing gene expression values along with the corresponding mean MES, mean EPITH, and MES/EPITH ratio across samples of the same cell type.
4. Embryonic origin annotations were assigned to each cell type.
5. A Pearson correlation matrix was generated across collapsed cell types, displaying annotation data related to embryonic origin, mean MES, mean EPITH, and the MES/EPITH ratio.

### 2.5. Differential Expression Analysis

Differential expression analysis comparing groups of different embryonic origins was performed using DESeq2. The gseGO algorithm (from the clusterProfiler package [14]) was applied to the gene list ranked based on DESeq2-provided statistics (Wald test values) to identify Gene Ontology terms significantly enriched or suppressed in the experimental groups.

*p*-values were adjusted using the Benjamini-Hochberg (BH) correction to control the False Discovery Rate (FDR), resulting in adjusted *p*-values (padj).

### 2.6. Bar Plots and Statistics

The mean expression values of MES, EPITH, and the MES/EPITH ratio for samples grouped by cell type in the GTEx-derived dataset were displayed as bar plots. Statistical significance of differences between groups was calculated for each plot using a one-way ANOVA model.

## 3. Results

### 3.1. Pre-Implantation Embryo Displays a Predominant Mesenchymal Transcriptional Phenotype

**Figure 1:**
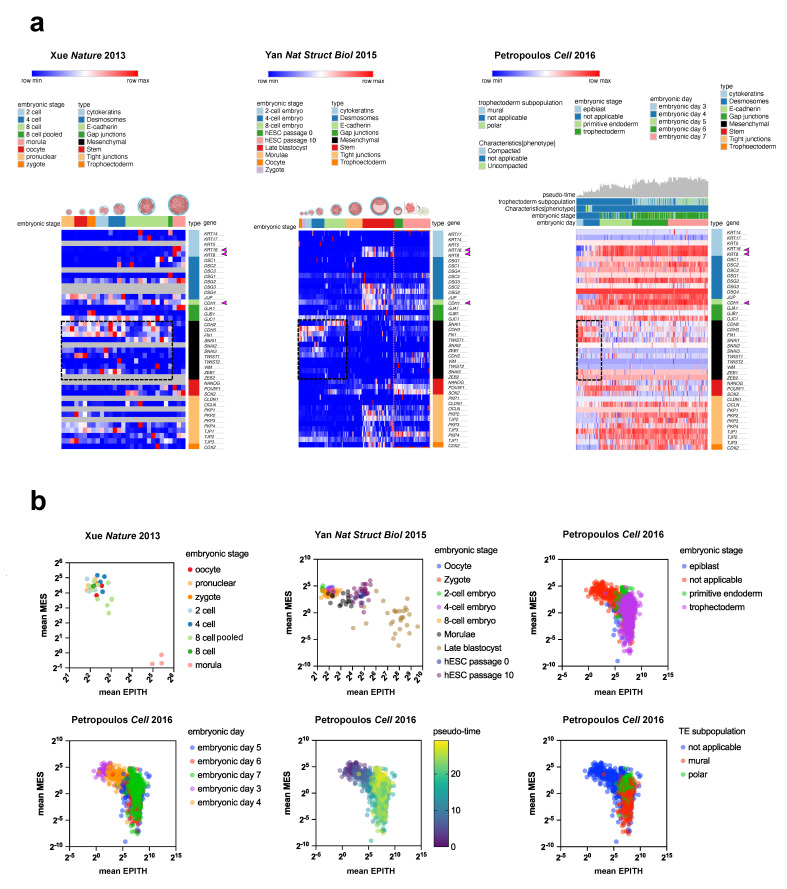
Heatmaps of three distinct but partially overlapping single-cell RNA-seq datasets showing that early pre-implantation embryo stages display an upregulation of mesenchymal markers compared to the 8-cell stage, blastocysts, and trophoectoderm (**a**). The color scale reflects the relative maximum and minimum values across samples (rows) in each dataset. The mean values of mesenchymal (mean MES) and epithelial (mean EPITH) genes are shown for each embryonic stage, embryonic day, pseudo-time, or trophoectoderm (TE) subpopulation, as indicated (**b**). “NA” indicates samples for which no annotation is provided in the corresponding paper. Pink arrowheads on the side of the heatmaps indicate the epithelial markers indicated in the text.

Before specific molecular markers were identified, the classical parameters for distinguishing epithelial and mesenchymal phenotypes relied on apico-basal polarity and tight junctions (zonula occludens) that seal the extracellular space [1,2,3,4,5,6,7,8,15]. In mice, blastomeres exhibit a round, non-polar morphology with loose or absent cell–cell connections from the zygote to the eight-cell stage [16]. At the eight-cell stage, blastomeres start compacting, gap junctions appear, and adherens junction components (E-cadherin and catenin), previously accumulated, begin functioning as an integrated adhesion system [15,17]. However, comprehensive profiling of epithelial and mesenchymal markers in early mammalian embryogenesis is still lacking. To address this, we analyzed three publicly available single-cell RNA-seq human datasets [9,10,12] for the expression profile of genes associated with the mesenchymal phenotype and junctional systems (Figure 1). Heatmaps were generated, normalizing genes across rows to show relative expression between samples (Figure 1a). All three datasets consistently showed relative overexpression of mesenchymal genes (e.g., *SNAI3*, *CDH2*, *CDH3*, *ZEB1*, *FN1*, and *TWIST1*) at early stages, declining after the eight-cell stage and giving way to epithelial genes (e.g., *CDH1*, *KRT8*, and *KRT18*) and tight junction genes at the late blastocyst stage (Yan et al. dataset [10]) and in the trophoectoderm (Petropoulos et al. dataset [12]) (Figure 1a). Furthermore, embryonic stem cells extracted from the inner cell mass of the blastocyst (Yan et al. dataset, orange dashed square) lacked *KRT8* and *KRT18* expression, showed slightly elevated mesenchymal gene expression, and had reduced epithelial-tight junction gene expression compared to the whole late blastocyst. The scatterplot of median absolute expression values for mesenchymal vs. epithelial markers (Figure 1b) highlighted a predominant mesenchymal phenotype with a detectable level of epithelial markers, which peaked at the eight-cell stage before mesenchymal markers declined at the blastocyst stage. These data suggest that the pre-implantation embryo displays traits of a mesenchymal phenotype, which quickly shifts toward a fully epithelial state with cell compaction and TE formation, challenging the traditional view that epithelial tissue is the first determined histotype and that mesenchyme appears later as an epithelial derivative.

### 3.2. Epiblast Displays an Intermediate Phenotype Between Mesoderm and Ectoderm/Endoderm

**Figure 2:**
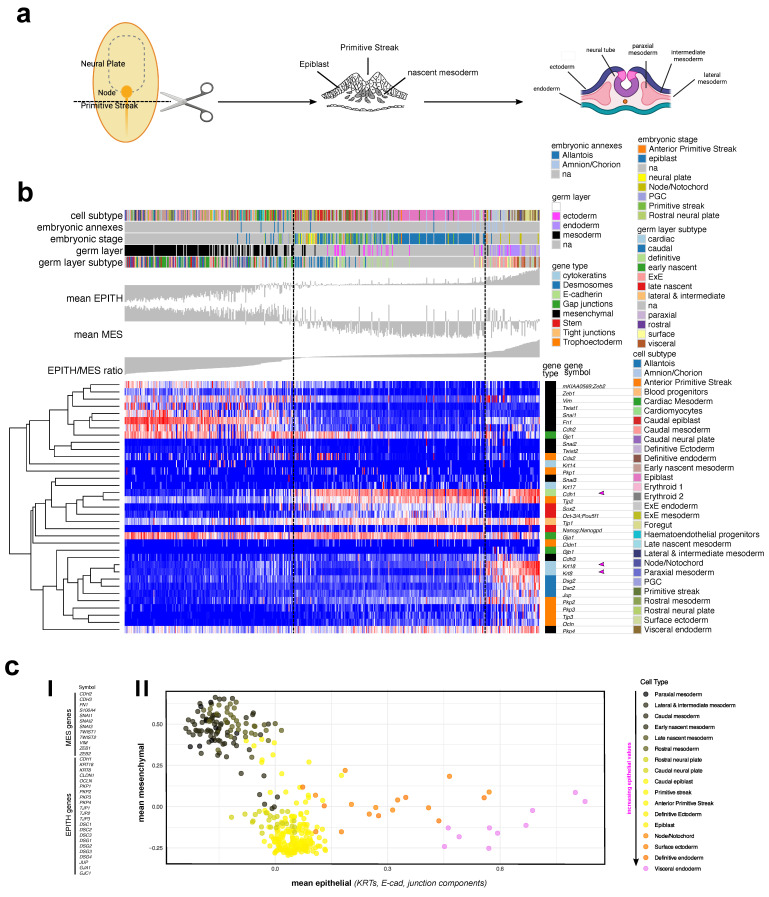
The ingression of epiblast cells into the primitive streak leads to the formation of the three germ layers during gastrulation (**a**). A heatmap shows the relative expression values of mesenchymal and junction-related genes from the Mittnenzweig dataset [11], which includes samples from multiple intermediate stages from blastocyst to gastrula (**b**). Annotations for samples across multiple grouping parameters provided by the authors (embryonic annexes, embryonic stage, germ layer, and germ layer subtypes) are shown. For each sample, mean MES, mean EPITH, and the mean MES/EPITH expression ratio are displayed as bars above the heatmap (**b**). The values of MES and EPITH genes listed in (**cI**) were calculated for each group and plotted. Colors were assigned to each group on a black–yellow–pink gradient scale according to the mean MES/EPITH ratio within the group (**cII**). “NA” indicates samples for which no category is assigned in the corresponding paper.

At the late blastocyst stage, the inner cell mass forms the epiblast and the hypoblast (also known as primitive endoderm) (Figure 2a). Traditionally, the epiblast is described as an epithelial sheet, and its transformation to form mesoderm is considered the prototype of EMT [1]. Analysis of the Mittnenzweig mouse dataset [11] (Figure 2b) shows that the epiblast (cyan in the embryonic stage color bar) has low expression of both mesenchymal and epithelial markers, except for *Cdh1*, compared to its derivative layers, mesoderm and endoderm (black and pink, respectively, in the germ layer color bar). The mesoderm shows a distinctly high mesenchymal phenotype, the endoderm an epithelial phenotype, and ectoderm and neural plate (yellow in the embryonic stage color bar) a slightly higher expression of mesenchymal markers than the epiblast. Notably, the primitive streak, an intermediate phase between the epiblast and mesoderm and a prototype of EMT, exhibited a slightly higher expression of mesenchymal markers than the epiblast, though much lower than the mesoderm. The endoderm, a prospective source of epithelial tissue for inner organs, displayed markedly higher epithelial marker expression (mainly *Krt8* and *Krt18*) (Figure 2b, pink arrowhead) than the epiblast, ectoderm, and neural plate, while still expressing mesenchymal markers (mainly *Vim*, *Fn1*, and *Cdh2*) (Figure 2b), indicating a hybrid E/M phenotype.

To simplify the visualization of the mesenchymal/epithelial profile of different embryonic stages and structures, mean expression values for mesenchymal (MES) and epithelial (EPITH) genes (as defined in Figure 2cI) were plotted against each other, with colors assigned according to the mean EPITH expression (Figure 2cII).

### 3.3. Mesenchymal/Epithelial Profile of Different Adult Cell Types Reflects Embryonic Origin

**Figure 3:**
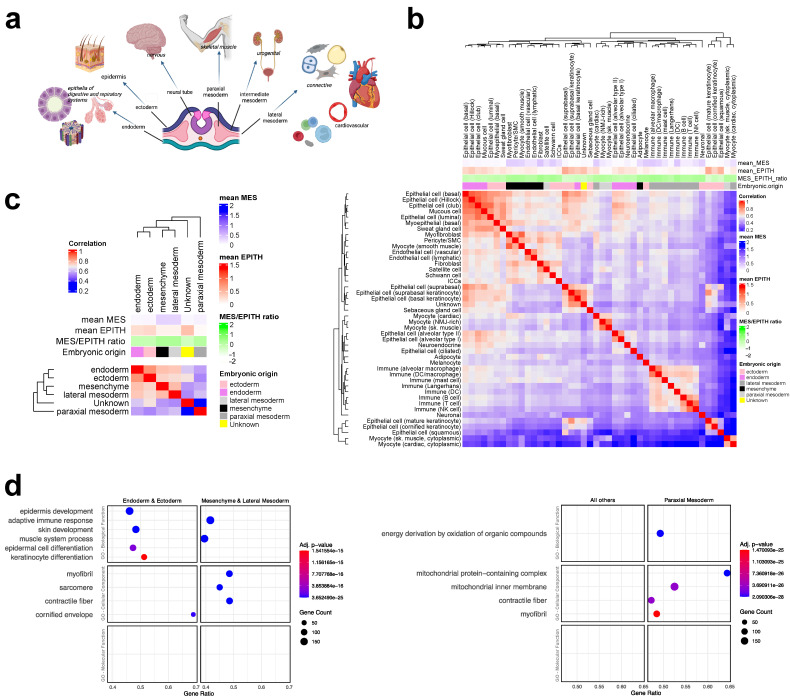
The three germ layers in the gastrula give rise to all tissues and organs of the body (**a**). A Pearson correlation matrix was generated based on the whole transcriptome of a GTEx-derived dataset, which includes single-nucleus RNA-seq transcriptomic profiles of 209,126 cells from 25 samples across 8 human adult organ types [13]. Metadata were implemented with annotations for “embryonic origin” and the calculation of mean MES, mean EPITH, and the MES/EPITH ratio for samples collapsed either by cell type (**b**) or by embryonic origin (**c**). DESeq2 analysis, followed by gseGO analysis with stat (Wald test)-based ranking of genes, was performed to identify differentially expressed genes between the indicated pairs. The dot plots display only the 10 most significant GO terms from the gseGO analysis across the three GO database subcategories: Biological Process (BP), Cellular Component (CC), and Molecular Function (MF) (**d**).

During gastrulation, the epiblast forms three germ layers that give rise to all tissues and organs (Figure 3a). Broadly, ectoderm generates epidermis and the nervous system, endoderm generates the epithelial lining of the respiratory and gastrointestinal tracts, and mesoderm generates muscle, urogenital, and cardiovascular systems, as well as all connective tissues via mesenchyme.

To assess how the embryonic origin relates to the general transcriptome of adult tissues and their MES and EPITH profiles, we analyzed data from the GTEx project database (https://gtexportal.org/home/). This database provides whole-genome single-nucleus RNA-seq profiles of 209,126 cells from 25 samples spanning eight human adult organ types [13]. Original metadata were supplemented with the mean values of MES and EPITH genes (as defined in Figure 2cI), as well as MES/EPITH ratio values for each sample. The gene expression matrix along with the corresponding values of MES, EPITH, and MES/EPITH was reduced by collapsing samples according to their cell type annotation, as provided by the original metadata. Afterwards, a prospective embryonic origin was assigned to each cell type.

The correlation matrix of the whole transcriptome across cell-type-collapsed samples (Figure 3b) revealed two major clusters within which samples are grossly grouped, based on their embryonic origin and MES/EPITH expression profiles. This suggests that embryonic origin and MES/EPITH profiles strongly relate to the general transcriptome in adults. Notably, cell types derived from lateral and paraxial mesoderm were segregated in different clusters, thus suggesting a major difference in their transcriptomes. The correlation matrix of samples collapsed by embryonic origin (Figure 3c) indicated a slight hierarchy in similarity between samples derived from different germ layers. Specifically, samples from endoderm and ectoderm as well as mesenchyme and lateral mesoderm exhibited greater similarity to each other. In contrast, samples derived from paraxial mesoderm were more distinct. Differential expression analysis (DESeq2) and subsequent Gene Set Enrichment Analysis (GSEA, via gseGO) identified mitochondrial genes as the most defining feature of tissues derived from paraxial mesoderm (e.g., skeletal muscle) compared to other tissues.

**Figure 4:**
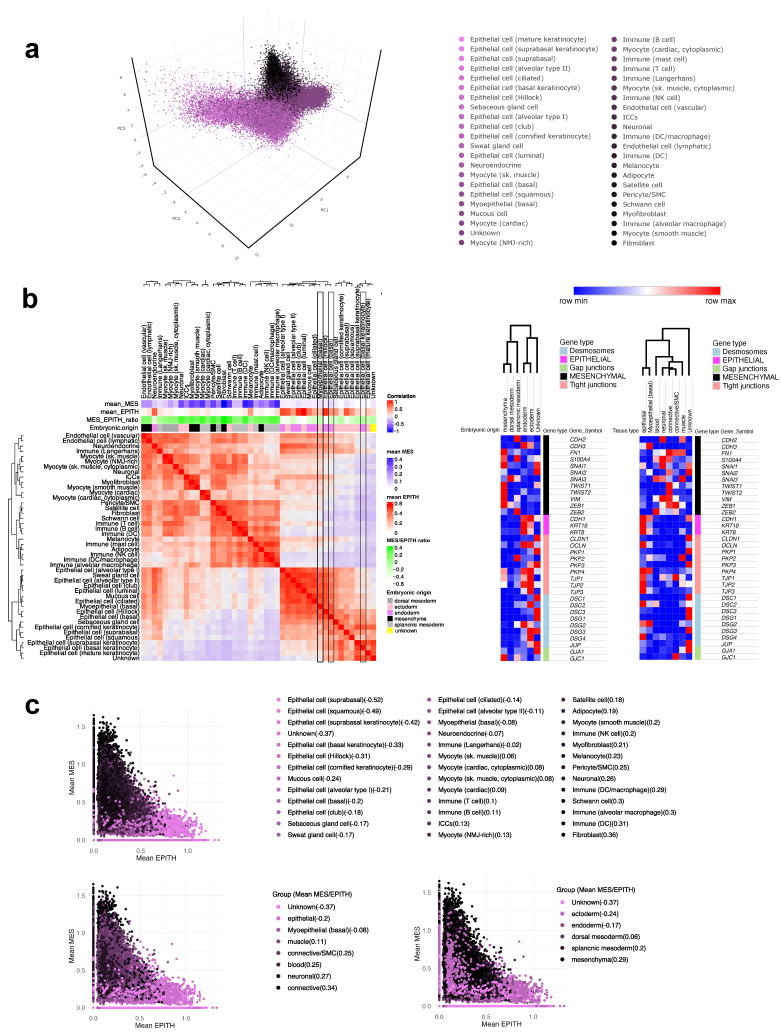
The expression profiles of MES and EPITH genes in cells from the GTEx-derived dataset were separated in PCA space (**a**). Different colors along a scale from pink to black were assigned to each sample based on the mean K-means clustering value of all samples belonging to the same cell type. A Pearson correlation matrix and hierarchical clusterization heatmaps were generated to assess whether the expression profile of MES and EPITH genes might segregate sample groups of different embryonic origin and/or tissue types (**b**). Boxes highlight the meanMES, meanEPITH, and MES/EPITH ratio of varied population of epithelial basal cells within the correlation matrix (**b**, left panel). expression values of MES and EPITH genes were plotted, with colors ranging from pink to black assigned according to the mean MES/EPITH ratio for each cell type, embryonic origin, or tissue type, as indicated (**c**).

To more closely evaluate the relationship between cell types, embryonic origin, and expression profiles of MES and EPITH genes, varied approaches of analyses were performed on the restricted subset of MES and EPITH genes (as defined in Figure 2cI). Samples were initially visualized in a 3D plot based on PCA (five dimensions). A k-means algorithm (two clusters) was applied to classify samples by PCA profile, with a color gradient from pink to black representing each cell line according to the average k-means value. This gradient reflects spatial proximity in the PCA plot (Figure 4a). Epithelial and connective cells were generally separated, while myocytes and blood cells were intermediate. A correlation matrix was then generated, with annotations for embryonic origin, mean expression values of MES and EPITH genes, and the MES/EPITH ratio (Figure 4b). The MES and EPITH profiles mirrored embryonic origin: mesoderm- and mesenchyme-derived samples showed a predominant mesenchymal phenotype, while endoderm- and ectoderm-derived samples were epithelial. Remarkably, basal cells of epithelial tissues exhibited a slightly higher MES/EPITH ratio compared to most other epithelial populations (Figure 4b, black boxes). Finally, MES and EPITH values were plotted for each sample, with colors assigned according to the MES/EPITH ratio of each sample’s cell type, tissue type or embryonic origin (Figure 4c).

### 3.4. The Basal Cells of Adult Epithelial Tissues Retain a Hybrid E/M Phenotype

**Figure 5:**
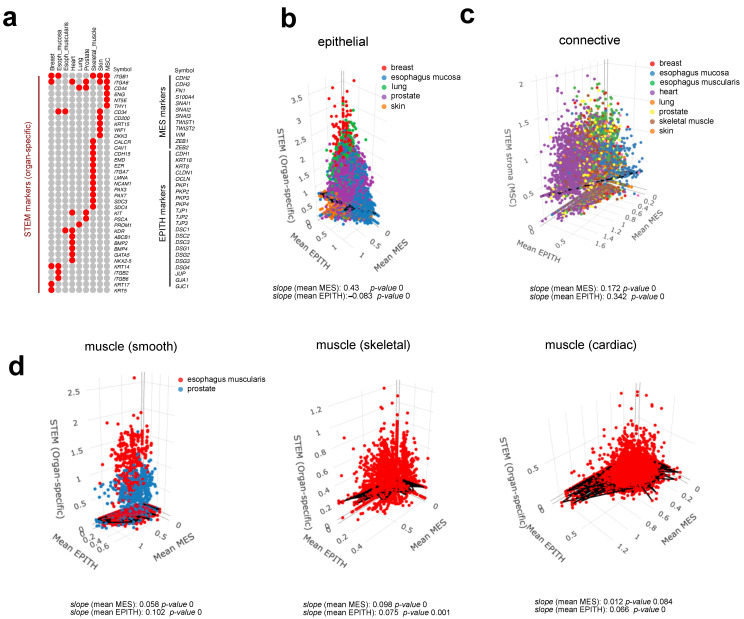
A stem cell score (STEM organ-specific) was assigned to each sample in the GTEx-derived dataset [13], calculated as the mean expression value of organ-specific marker panels as indicated in the figure (**a**), depending on the tissue type and organ each sample belonged to. The STEM organ-specific score for each sample was plotted against the mean MES and EPITH gene expression values for each tissue type, as indicated (**b**–**d**). Linear regression fitting for each plot was calculated using the Ordinary Least Squares (OLS) model.

Dong et al. [8] recently demonstrated that epithelial tissue precursors during organogenesis predominantly display a hybrid E/M state resembling intermediate epithelial/mesenchymal cells in tumorigenesis. Adult tissues retain populations of stem cells with restricted differentiation potential. To evaluate whether these stem cell populations retain a hybrid E/M phenotype, we calculated a stem cell score (STEM organ-specific) for each cell within the respective tissue and organ based on organ/tissue-specific stem cell marker panels (Figure 4a, left panel). Mean expression values of STEM organ-specific markers for each cell were plotted against mean MES and EPITH marker (Figure 5a, right panel) expression (Figure 5b–d).

The STEM organ-specific signature in epithelial cells correlated positively with MES but negatively with the EPITH phenotype, indicating that adult epithelial precursors display traits of a mesenchymal phenotype and undergo a mesenchymal-to-epithelial transition (MET) upon terminal differentiation (Figure 5b). In contrast, STEM organ-specific markers correlated positively with both MES and EPITH markers in muscle and connective tissue cells (Figure 5c,d).

**Figure 6:**
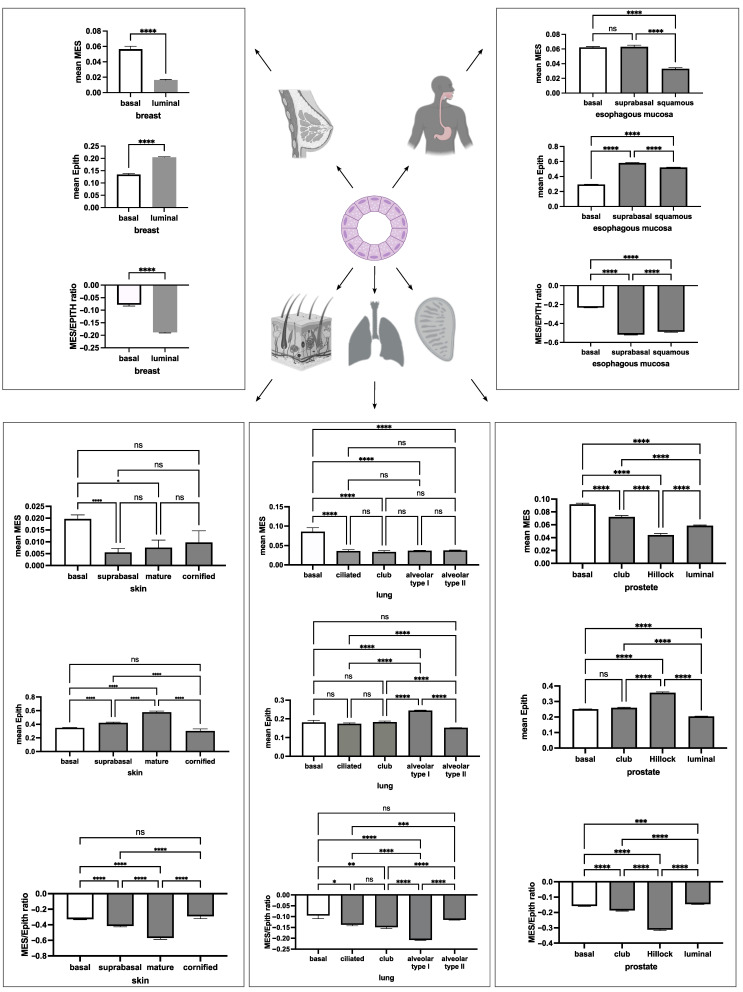
The mean (+/− SE) expression values of MES, EPITH, and the MES/EPITH ratio for epithelial samples in the GTEx-derived dataset were grouped by cell type according to the annotations provided by the authors and displayed as bar plots (mean + SE). Statistical significance of differences between groups was calculated for each plot using a one-way ANOVA model. ns: not significant; * *p* < 0.05; ** *p* < 0.001; *** *p* < 0.0001; **** *p* < 0.00001.

**Figure 7:**
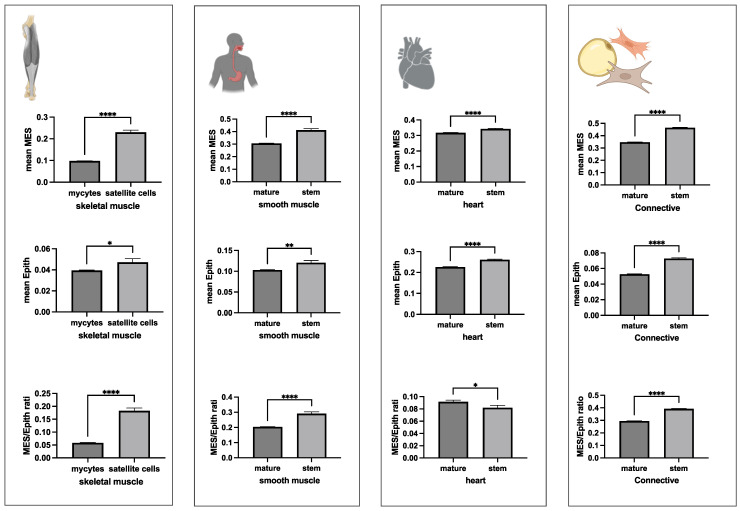
The mean (+/− SE) expression values of MES, EPITH, and the MES/EPITH ratio for skeletal muscle samples in the GTEx-derived dataset were grouped as either stem (satellite cells) or mature, based on annotations provided by the authors, and displayed as bar plots. The mean expression values of MES, EPITH, and the MES/EPITH ratio for either cardiac muscle, smooth muscle, skeletal muscle, or connective tissue cells in the GTEx-derived dataset were grouped as either stem or mature, based on the positivity (mean value > 0) for the corresponding panel of STEM organ-specific markers indicated in Figure 4a. Statistical significance of differences between groups was calculated using one-way ANOVA. ns: not significant; * *p* < 0.05; ** *p* < 0.001; **** *p* < 0.00001.

These results were further evaluated using an alternative data analysis approach, consisting of a statistical comparison of the MES, EPITH, and MES/EPITH ratio values between basal/satellite cells and their mature counterparts, as annotated in the original dataset paper [13], whenever available. Since the original paper does not provide annotation of the stem precursors of smooth muscle cells, cardiomyocytes, and connective tissue cells, for these cell types, stem and mature cells were identified based on the positivity (mean value > 0) or negativity (mean value < 0) of the STEM organ-specific markers indicated in the left panel of Figure 5a. Consistent with the previous analysis, basal epithelial cells displayed higher MES expression and lower EPITH marker expression compared to most of their mature counterparts (Figure 6), whereas stem precursors of muscle and connective tissue cells exhibited higher expression of both MES and EPITH markers (Figure 7) than their mature counterpart.

## 4. Discussion

Traditionally, epithelial tissue is considered the first clearly determined tissue type to emerge in early embryogenesis, forming structures such as the trophectoderm, epiblast, and primitive endoderm. The mesenchymal phenotype is thought to appear later through EMT processes. The prototype of EMT is the migration of epiblast cells through the primitive streak to form primary mesenchyme [1], which subsequently undergoes partial re-epithelialization to generate extraembryonic, paraxial, intermediate, and lateral mesoderm [2]. The mesoderm, with additional contributions from neural crest cells derived from the cranial ectoderm, undergoes a further round of EMT to form secondary mesenchyme, historically termed embryonic connective tissue [18], which serves as the progenitor for fibroblasts, osteoblasts, chondroblasts, and adipocytes.

The conceptual evolution of “mesenchyme” as an EMT derivative during gastrulation has contributed to the notion that epithelium is the default tissue of the body, while the mesenchymal phenotype is seen as a transient state between epithelial stages or as a terminally committed connective tissue lineage. This traditional view has also influenced interpretations of pathological EMT, particularly its aberrant activation in cancer. While carcinomas are, by definition, epithelial, the acquisition of mesenchymal traits in carcinoma cells has long been associated with aberrant reactivation of an EMT program that promotes invasion and metastasis [19].

Recent evidence in cancer biology has disrupted the traditional dichotomy between epithelial and mesenchymal phenotypes, demonstrating a continuum with hybrid epithelial/mesenchymal (E/M) states (*quasi-mesnechymal* states) [6]. These hybrid E/M states are characteristic of cancer cells with stem cell properties and enhanced aggressiveness. Although a quasi-mesenchymal phenotype has also been reported in normal stem cells, particularly in breast tissue [4,6], it is generally perceived as a partial or incomplete EMT activation occurring in cancer progression. Little is known, however, about the role of these hybrid states in the continuum from prenatal to postnatal development.

Our findings indicate that hybrid E/M phenotypes are general features of undifferentiated cells. The pre-implantation embryo expresses a range of mesenchymal markers, which are gradually replaced by epithelial markers between the four- and eight-cell stages, coinciding with trophectoderm formation. This observation suggests that the mesenchymal phenotype may represent the default cellular state in the body, with the first epithelial tissue (trophoectoderm) derived from a mesenchymal-to-epithelial transition (MET) event in early embryos. The epiblast, typically viewed as an epithelial sheet, expresses markedly lower levels of epithelial markers than the ectoderm and endoderm, its plasticity-restricted derivatives.

The formation of the three-germ layers at gastrulation consists of the acquisition of either MES (mesoderm) or EPITH profiles (endoderm and ectoderm) that are globally maintained in their adult derivatives, as expected. However, within the epithelial tissues, the basal cells display a slightly higher MES/EPITH ratio.

Dong et al. [8] recently demonstrated that epithelial tissue precursors during organogenesis exhibit a predominantly hybrid E/M state, similar to intermediate epithelial/mesenchymal cells in tumorigenesis. The findings presented here suggest that these hybrid E/M phenotypes persist in adults, particularly within basal precursors of epithelial tissues, implying that these hybrid E/M states may represent transitional developmental stages that span the adult life in a *standby mode* as soon as it is needed.

The conceptual link between full-epithelial phenotypes and a terminal differentiation state aligns with the architectural, morphological, and physiological constraints that epithelial tissues must fulfill in the adult organism. Epithelial tissues cover external body surfaces and line organ cavities, functioning as specialized interfaces that facilitate complex interactions with the external environment, selective nutrient absorption, and local or systemic release of targeted molecules. These functions necessitate a high degree of functional specialization and terminal commitment.

Collectively, these data support the notion that the mesenchymal phenotype and/or the hybrid E/M states might hallmark undifferentiated states. The conclusions of this study are based on the expression of a selected group of epithelial and mesenchymal markers. Since cellular states are often influenced by complex regulatory networks beyond these markers, further studies are needed to explore how the integration of mesenchymal and epithelial features might impact on the cell function and stem cell biology in pathological and physiological contexts.

## Data Availability

Original source and accession numbers of the dataset used in this work are detailed in the Section 2.
